# Time to initiate randomized controlled clinical trials with methadone in cancer patients

**DOI:** 10.12688/f1000research.20454.1

**Published:** 2019-10-31

**Authors:** Hans-Joachim Kremer

**Affiliations:** 1 Medical Writing Service, Freiburg, 79117, Germany

**Keywords:** Methadone, doxorubicin, cancer, randomized controlled clinical trials

## Abstract

Public media coverage has fueled a demand for methadone as potential cure for cancer itself. Because patients have asked for respective prescriptions, clinical societies issued statements warning against the use of methadone as long as preclinical findings have not been supported by clinical evidence. In fact, not all preclinical data clearly support relevant effects. However, strong epidemiologic data suggest beneficial effects of methadone on cancer. Alternative explanations, namely better safety of methadone or hidden selection bias, seem less likely. This uncertainty can only be resolved by randomized controlled clinical trials. This review discusses all relevant data pertinent to methadone and cancer, uncovers supportive epidemiologic data, and suggests possible study designs.

## List of abbreviations

AIDS         Acquired immunodeficiency syndrome

ART          Antiretroviral therapy

CI              Confidence interval

CNS          Central nervous system

HIV           Human immunodeficiency virus

HR            Hazard ratio

MGMT     O6-methylguanin-DNA-methyltransferase

MMT        Methadone maintenance therapy

OR           Opioid rotation

SD            Standard deviation

VA            Veterans Affairs

## Background

Since 2017, public media have suggested methadone as a potential cure of cancer. As a result, patients are demanding a prescription of methadone when cancer has been diagnosed or has progressed, perhaps with pain as an excuse. An unrepresentative online survey among German oncologists, conducted in summer 2017, indicated that 83% of 473 responding physicians had experience with patients who “frequently or very frequently” asked for methadone therapy ^1^. There are indications that an increase in the demand for methadone has spread from Germany to Austria and Switzerland, and beyond ^2, 3^.

It is certainly not in the interest of medical sciences and not of future patients, if today’s patients continue demanding methadone for an unproven therapy. Not surprisingly, several clinical societies have published warning statements on the use of methadone as a cancer therapy ^4– 7^. While all of these societies called for controlled clinical data, they did not evaluate encouraging data on methadone and did not propose designs for a clinical trial. Sometimes, they warned on use of methadone with questionable arguments on its safety. Whether associated to this skepticism or not, no clinical trial evaluating methadone for cancer has yet been initiated, although 152 and 47 trials with methadone as an intervention, but other uses, have been posted on clinicaltrials.gov and in the EU Clinical Trials register, respectively, since 2013 ^8^. Therefore, this article reviews the all data that could provide a rationale for evaluating methadone as an anticancer agent.

General pharmacological properties of methadone are not discussed here. These properties were discussed in a review proposing methadone as a “tumor theralgesic”. However, it did this without thoroughly discussing the clinical and epidemiologic evidence ^9^. It should be stressed that methadone is a racemate and should be understood as such unless therwise indicated. The levo- or (–) form is considered to be active at the µ-opioid receptor, the dextro or (+) form is considered inactive there; other opioid receptors appear to be largely unaffected by both forms ^10^. In the USA, methadone is licensed for the treatment of moderate to severe pain not responsive to non-narcotic analgesics, for detoxification treatment of opioid addiction, and for maintenance treatment of opioid addiction ^11^. In many European countries, methadone is only approved for the treatment of opioid drug addiction ^12^. In some European countries, levomethadone is available for the treatment of severe pain and treatment of opioid drug addiction ^13^. Dextromethadone has not been approved anywhere, but is currently undergoing a clinical research program major depressive disorder ^14^.

## Preclinical findings

Preclinical data, rather than clinical observations, have triggered research on the use of methadone in cancer. Already in the 1980s, studies on neuroblastoma indicated delayed tumor growth after opioids of the morphine-type and their antagonism by naltrexone ^15– 17^. Similar findings were reported for morphine and naloxone in lung cancer cells, although in concentrations far above the therapeutic range ^18^. That group then investigated methadone in human lung cancer cells ^19^. Some effects were observed already at concentrations of 1 nM and strong effects at 10 nM; 10 nM correspond to about 3 ng/mL, i.e. rather below therapeutic plasma levels (see below). They also found similar effects with dextromethadone alone, which was slightly more active than levomethadone in some cell lines. These data prompted further studies by this group confirming the effects of methadone ^20– 23^. Apparently, these findings were not challenged by other groups.

Rather high concentrations of methadone, but not morphine, hydromorphone, or naloxone, increased the accumulation of vinblastine in multidrug resistant cells ^24^.

Interest in the antineoplastic potential of methadone was revived about 10 years later when a German group around Claudia Friesen took up these data ^25^. Her group found that methadone inhibited proliferation of HL-60 myeloid leukemia cells and activated apoptosis pathways. In particular, methadone was able to overcome chemo- and apoptosis resistance, especially but not only resistance to doxorubicin (HL-60 cells were resistant to doxorubicin at a concentration of 100 ng/mL). Methadone alone showed some effects at 15 µM (5000 ng/mL) and was fully effective at 30 µM. Note, however, that peak concentrations of methadone after oral administration in humans were found between 124 to 1255 ng/mL, and steady state levels between 65 to 630 ng/mL ^11^. Hence, the effects in these cell lines might be insufficient for claiming antineoplastic effects of methadone alone under therapeutic conditions.

Then a Spanish group found that methadone was able to induce a necrotic-like cell death in SH-SY5Y cells, i.e. a cell line commonly used in basic research on neuronal functions and cancer ^26^. Although this group admitted to having observed these effects in supratherapeutic doses, they concluded that their finding could explain the toxic effects on various cell lines including cancer and leukemia cells. A Canadian group found methadone to induce apoptosis in pediatric acute lymphoblastic leukemia cells ^27^. They determined high levels for IC _50_ for all but only one pro-B-cell leukemia cell line that exhibited an IC _50_ of “only” 9400 ng/mL, i.e. still clearly above the therapeutic range.

In 2008, the German group filed a patent application that claimed, among others things, that methadone (1000 ng/mL) could enhance the apoptotic effects of doxorubicin (100 ng/mL) in glioblastoma cells ^28^. Corresponding details were published 2013 showing that methadone at a concentration as low as 100 ng/mL was able to enhance the apoptotic effects of doxorubicin in concentrations between 10 and 60 ng/mL in acute lymphoblastic leukemia cells ^29^. Peak plasma concentrations of doxorubicin average 4100 ng/mL (SD 220) after a modest 10 mg/m ^2^ dose ^30^. Thus, this apoptosis-enhancing combinatorial effect might occur in the therapeutic range of both substances. The authors explained the interaction by increased cellular uptake of doxorubicin by methadone, which in turn might be explained by a down-regulation of cAMP by the opioid ^29^. This group then investigated this interaction in glioblastoma cell lines and confirmed previous findings on apoptosis and doxorubicin enhancement ^31^. In this setting methadone exhibited significant effects at 1000 ng/mL or higher.

Thereafter, public interest grew, fueled by public media; the above-mentioned critical statements from oncologic societies were published, and methadone was investigated by other groups. Levomethadone was found to be ineffective in glioblastoma cells; however, data on the racemate were not reported by that group ^32^. Instead of doxorubicin they used temozolomide, an alkylating agent that is recommended for glioblastoma, in contrast to doxorubicin, which is unable to cross the blood-brain barrier, and therefore not recommended for glioblastoma ^30^. In fact, no controlled clinical trial of doxorubicin in glioma has ever been published. Conversely, it is unclear whether temozolomide as such is useful *in vitro*, as it is active only after conversion to 5-(3-methyltriazen-1-yl)-imidazole-4-carboxamide (MTIC) ^33^. Another group also found no interaction of methadone and temozolomide in glioblastoma cell lines and methadone being active in only one cell line at a high concentration of 15 000 ng/mL ^34^.

While methadone alone was inactive in melanoma cell lines from a biobank, its combination with cisplatin decreased viability in a cell line displaying a high expression of OPRM1, a main receptor for methadone ^35^. However, cell lines expressing OPRM1 were rather rare in this biobank of melanoma cells.

A very recent report again denied *in vitro* interaction between methadone and temozolomide in glioblastoma cells, but reported decreased viability of fibroblasts after 1 µM methadone, i.e. about 300 ng/mL ^36^.

A more detailed review on preclinical research findings on antineoplastic effects of methadone was published end of 2018 ^3^.

## Epidemiological data

From the plethora of epidemiologic studies with methadone, those with data on mortality were selected.

### Rather normal, non-HIV, non-metastatic patients

A thorough analysis of the Veterans Affairs (VA) database compared mortality after treatment with long-acting morphine and methadone ^37^. The aim was to compare the safety as some authors had suggested disadvantages of methadone.

The authors identified patients who had received a new prescription for either methadone or long-acting morphine. The time frame was 2000 through 2007. They analyzed 98,068 patients in the primary analysis cohort. It should be stressed that they specifically excluded patients diagnosed with metastatic cancer and those in palliative care, and patients receiving methadone for opioid addiction (because morphine is not used for this indication and would therefore be an inappropriate control). The analyzed population was composed of patients who mainly used the drugs for a non-cancer pain indication and some who used them for non-metastatic cancer pain; less than 2% had HIV/AIDS ( Table 1).

**Table 1.** VA study of Krebs *et al.* 2011: Main data of the propensity quintiles.

Quintile	1		2		3		4		5	
	Meth	LA mo	Meth	LA mo	Meth	LA mo	Meth	LA mo	Meth	LA mo
P·Y	947	5 707	2 923	11 775	4 202	11 739	5 869	10 989	8 179	8 268
Ratio Me/Mo	0.166		0.248		0.358		0.534		0.989	
Age (mean, y)	64	64	60	61	58	58	55	55	52	52
Any malign. (%)	43	45	29	30	17	17	10	10	4	4
MI (%)	14	13	13	13	10	10	8	8	6	6
CHF (%)	25	24	21	21	17	17	14	14	10	10
HIV/AIDS (%)	2	2	2	2	1	1	<1	<1	<1	<1
Tobacco disorders [%]	36	34	41	39	44	44	48	48	52	53
Back pain (%)	63	59	74	74	81	82	88	89	94	93
Joint or limb pain (%)	79	74	81	82	85	85	88	87	89	90
HR	0.36		0.48		0.50		0.66		0.92	

P·Y: Person-years ^37^. Ratio Me/Mo indicates the ratio of the P·Y values, methadone by long-acting (LA) morphine per quintile. The background data were derived from the supplementary appendix ^37^. The upper 2 lines (age, any malign.) show two variables that presumably triggered the advantage for methadone over LA morphine concerning survival. The middle 4 lines (MI, CHF, HIV/AIDS, Tobacco) show otherwise important variables related to survival without obviously relevant differences between the groups. Back pain and joint limb pain are shown as main explanation for the indication and the almost turned Me/Mo ratio in Quintile 5. An HR below 1 indicates a lower mortality after methadone compared with LA morphine, the lower the better. The HR of 0.36 could be translated e.g. into 100/1000 LA morphine patients died but only 36/1000 methadone patients in the same time interval. MI: Myocardial infarction. CHF: Congestive heart failure. Note that the paper did not provide data on the body mass index.

To control for selection bias, they calculated propensity scores based on several demographic and medical data and split the set into five demographically more homogenous quintiles. The Kaplan-Meier survival function calculated for these quintiles found clearly improved survival (i.e. low hazard ratios, HR, cf. Table 1) in Quintile 1 favoring methadone over long-acting morphine. Quintiles 2 to 4 also indicated an advantage of methadone, but less clearly. The last quintile indicated no difference in survival. The actual survival curves indicated that Quintile 1 was at greatest risk, while Quintiles 4 and 5 were at lowest risks. A closer look at the data including the appendix of this article ^37^ suggests that “any malignancy” was the most important dichotomic factor and that age was the most important continuous factor yielding low propensity scores and lower quintile numbers.

To test the robustness of their findings, the authors also analyzed the data after exclusion of patients “with any cancer diagnostic code”; note that this term led to numbers different from those pertinent to “any malignancy”. The authors found that the difference in survival in this subcohort “noncancer” was reduced as compared to the primary analysis set ( Table 2). These data allow a recalculation of the HR of the alternative subcohort “cancer”, which was even more favorable for methadone ( Table 2).

**Table 2.** VA study of Krebs *et al.* 2011: HR and 95% CI.

	N	Lower margin	HR	Upper margin	CI width
Primary cohort	98 068	0.51	0.56	0.62	0.11
Subcohort „non-cancer“	18 013	0.69	0.78	0.87	0.18
Subcohort „cancer“ ^a^	80 055	(0.4375)	0.51	(0.5825)	0.145

HR: Hazard ratio of survival estimates. Values below 1 indicate lower mortality after methadone (compared with long-acting morphine) ^37^. ^a^ HR and CI were not reported in that paper. However, the HR can be estimated from the data given. For the CI of this subcohort a CI width between the other 2 cohorts was assumed. Note that this is a very conservative strategy given the much higher sample size in the subcohort “cancer”.

Hence, this powerful epidemiologic study indicates:

- Methadone appears to have a survival advantage (HR 0.56) compared with long-acting morphine in patients which are non-metastatic, non-HIV, and non-addicts.
- The advantage is reduced or even abolished in patients without malignancy and in younger patients.
- The advantage is even more pronounced in patients with a malignant history. This holds true overall (subcohort “cancer”: HR 0.51) and especially for those with highest prevalence of malignancies (Quintile 1: HR 0.36).

The authors concluded, conservatively, that they found no evidence of excess all-cause mortality after methadone compared with long-acting morphine. They did not emphasize a possibly protective or beneficial effect with respect to cancer, nor did they provide any rationale for the significant difference in the primary cohort. The vast database makes a chance finding unlikely, although hidden selection bias still cannot be ruled out. The propensity scoring controlled for selection bias to the extent possible, as did other analyses of that group ^38^.

### Observational study with four treatment cohorts

An earlier retrospective cohort study investigated the safety of newly prescribed methadone, extended-release oxycodone, extended-release morphine, and transdermal fentanyl from a US Medicaid database ^39^. This study received less attention than the article discussed above, presumably due to the smaller sample size (5,684, divided into four treatment cohorts) and the lack of consistent differences. Abstracted from *p*-values and focused on estimates, this study can be considered in line with the VA study discussed above. Among cancer pain patients, mortality rates were lower after methadone than after morphine, while the difference was less pronounced in noncancer pain patients ( Table 3), *i.e.* a pattern similar to that of the VA study ( Table 2). The authors also found a trend towards fewer emergency department visits or hospitalizations for methadone compared with morphine ( Table 3), although, in all, methadone caused more frequent overdose symptoms. Methadone was also numerically better than oxycodone and fentanyl in cancer pain patients, suggesting unique properties of methadone.

**Table 3.** Observational comparative 4 cohort study.

	Patients with cancer				Patients with non-cancer pain			
	N	HR	95% CI	p	N	HR	95% CI	p
	**Emergency department visit or hospitalization**							
Methadone	178	0.24	0.05–1.13	0.071	508	0.70	0.29–1.69	0.426
Oxycodone	339	0.68	0.27–1.72	0.411	447	0.52	0.22–1.23	0.138
Fentanyl TD	307	1.08	0.43–2.74	0.870	338	1.42	0.63–3.21	0.404
Morphine	471	Reference			734	Reference		
	**Mortality**							
Methadone	178	0.48	0.18–1.23	0.127	508	0.78	0.29–2.13	0.628
Oxycodone	339	0.74	0.46–1.21	0.226	447	0.98	0.45–2.14	0.961
Fentanyl TD	307	0.93	0.58–1.49	0.768	338	0.89	0.43–1.84	0.753
Morphine	471	Reference			734	Reference		

Transcribed from Tables 3, 4, and 5 of reference ^39^. Estimates from Cox Proportional Hazard Models. HR: Hazard Ratio. TD: Transdermal (patch)

### Patients with non-cancer pain

A retrospective cohort study analyzed data obtained between 1997 and 2009 from the Tennessee Medicaid ^40^. The authors included 32,742 sustained-release morphine recipients and 6,014 methadone recipients, and excluded patients with evidence of cancer or HIV infection. They counted only “out-of-hospital mortality given that opioid-related deaths typically occur outside the hospital”. This resulted in the rule that “patients in the hospital could not enter the cohort until 30 days after discharge”, i.e. early mortality was disregarded. With this dataset they found an HR for mortality of 1.46 (95% CI: 1.17–1.83), indicating an increased risk of death after methadone. The authors concluded that the increased risk of death observed for users of methadone, even for low doses, supports recommendations that it should not be a drug of first choice for non-cancer pain. The data seem to contradict the VA study regarding the risk in the subcohort “non-cancer”. This difference can be explained by the discounting of early deaths in the Tennessee study. Exploratory analyses showed that overdose was the most frequent cause of unbalanced mortality. It should be remembered that methadone is often used in liquid form which facilitates overdose.

### Patients with HIV

Several studies and one meta-analysis ^41^ have provided comparative mortality data on methadone maintenance therapy (MMT) in HIV patients receiving antiretroviral therapy (ART). The meta-analysis found significant heterogeneity among the studies analyzed. Two Chinese cohort studies were most powerful, one ^42^ was included in the above mentioned meta-analysis, the other ^43^ is more recent. Both studies indicated that an MMT program is better than withholding methadone; the latter study indicated that stopping opioid use entirely leads to the lowest mortality rates. The proportion of virological suppression showed only a weakly inverse correlation with mortality ^43^. Patients with HIV infection are still at risk for the development of AIDS, and often die from various cancers, including Kaposi's sarcoma, non-Hodgkin lymphoma, Burkitt's lymphoma, primary central nervous system lymphoma, and cervical cancer. Interestingly, doxorubicin is first-line treatment for Kaposi's sarcoma and second-line for some forms of leukemia. Although it may be speculative to conclude anticancer efficacy of methadone from these data, they are at least compatible with beneficial effects of methadone alone or in combination with doxorubicin. Further epidemiological research should address a potential interaction.

### Miscellaneous

Literature search for mortality, methadone, and Kaposi sarcoma or lymphoma did not yield meaningful data. The same holds true for searches for Kaposi sarcoma or lymphoma, and vinblastine or doxorubicin, and methadone.

## Data from clinical trials

Cochrane reviews on methadone in noncancer pain ^44^ and cancer pain ^45^ are available. Both meta-analyses were hampered by low to very low quality of the source studies. The review in noncancer pain assessed the quantity and quality of evidence as too poor to draw conclusions on efficacy or safety between methadone, placebo, other opioids, or other treatments ^44^. The more recent review in cancer pain concluded that methadone has similar analgesic benefits when compared with morphine, is cheaper in many countries, but might be more difficult to handle than morphine or transdermal fentanyl ^45^. They found no differences in safety or mortality. A recent overview on several Cochrane reviews confirmed these statements ^46^.

## Targeted investigations

The following articles explicitly referred to one of Friesen’s articles ^25, 29, 31^.

### Retrospective study in glioma patients

Friesen and clinicians from Berlin, Germany, published a retrospective study on a rather poorly defined set of glioma patients: In total, 27 patients were exposed to methadone at different stages of therapy; 13 were exposed soon after diagnosis and 12 of these 13 were classified as evaluable for efficacy ^47^.

These 12 patients had been diagnosed with a primary glioblastoma multiforme and all initially received surgical therapy, most with gross total resection. After surgery, all received temozolomide and methadone. Of the 12, five were MGMT negative, i.e. at high risk. Of these five, four achieved progression-free survival for 6 months, which according to the authors could be compared with a published survival rate of 40%. The remaining seven patients were MGMT positive, i.e. at low to moderate risk. All seven MGMT-positive patients achieved progression-free survival for 6 months, which compared favorably with a rate of 79% observed in that centre before methadone was used. These data would be compatible with some beneficial effects, but even negative effects cannot be ruled out due to the small sample size.

The authors also presented safety, progression, and survival data of all 27 patients exposed to methadone. These data cannot be interpreted for efficacy due to heterogeneous baselines and therapies. The authors concluded that methadone can be safely combined with standard glioma chemotherapy. No patient was treated with doxorubicin.

### Retrospective opioid-rotation study

A US group analyzed the data of consecutive supportive care outpatients of a tertiary cancer center ^48^. They identified patients who underwent opioid rotation (OR), and defined two cohorts: OR to methadone (76 patients) and OR to any other opioid (88 patients). They found median survival after OR to methadone of 3.75 months (95% CI: 2.3–6.46) and after OR to other opioids of 2.62 months (95% CI: 1.74–4.33). The difference was not statistically significant, however, the small sample sizes should be considered. Given the short survival times the difference, if true, appears to be substantial.

The authors also provided data on patients with a follow-up visit. However, these data cannot be interpreted reasonably due to attrition bias, namely early deaths after rotation to other opioids.

### Case reports

Social media are referring to many cases. These are ignored here as long as not published in scientific journals.

There were five isolated case reports recently discussed in an opinion article from an Austrian group that is skeptical on methadone for cancer ^2^. Only one of these cases (counted as number 5) may have been relevant, as it was on concomitant use of chemotherapeutics and methadone; unfortunately, the outcome of this case was not reported. The other four cases may be, if any, of little relevance to the efficacy of methadone, as it was administered without concomitant chemotherapy. All these cases, however, illustrate the dilemma for physicians confronted with a patient’s last hopes, as methadone was always prescribed on patient’s demand.

## Discussion of evidence

The preclinical data suggests that racemic methadone itself can inhibit the growth of human lung cancer cells ^19^, increase the uptake of doxorubicin into leukemia cells ^29^, and reduce viability of fibroblasts ^36^ at concentrations that could be achieved with normal doses. There is little rationale to consider glioma or other CNS cancers as useful indication of methadone or add-on treatment with doxorubicin.

Epidemiological data are encouraging, most of all the VA study ^37^. The four-cohort study ^39^ numerically supports the findings of the VA study, as well as the opioid rotation study ^48^. The study investigating out-of-hospital mortality ^40^ should not be considered contradictory due to selective counting of deaths and the focus on noncancer pain. The positive outcomes of HIV studies ^42, 43^ might be explained by both the beneficial effects on cancer and a diminished use of illicit drugs. However, use of illicit drugs had no relevant quantity in the other studies.

The two targeted investigations were too small, but were also compatible with beneficial effects ^47, 48^.

All in all, epidemiologic and clinical data consistently indicate that patients treated with methadone had a better prognosis than those treated with morphine, and that this difference was larger the higher the proportion of patients with cancer diagnosis was.

What could alternative explanations be? Is methadone simply safer than long-acting morphine? Other evidence ^49^, guidelines ^50, 51^, and market data ^52^ on opioids do currently not support this assumption. Furthermore, there is no pharmacologic or physiologic rationale explaining why such safety advantage could be confined to unselected cancer patients.

Or was there a hidden selection bias? In fact, the VA study ^37^ did not control for socioeconomic status. However, it would be counterintuitive to assume that the “cheaper” methadone would be more often prescribed to the “rich” and the “more natural” morphine rather to the “poor”.

A reasonable explanation is that methadone yields protective or beneficial effects in cancer. And this effect cannot be explained by an effect of methadone in glioma or other central nervous cancers, as these cancers have too low a prevalence.

## What should be done?

If methadone is beneficial, whether alone or with doxorubicin, these effects may not be so overwhelming that historically controlled or other cohort studies would be sufficient to demonstrate efficacy. Any uncontrolled distribution of methadone is certainly inappropriate and should not be encouraged. Even prospective registry studies do not appear to be reasonable solutions, as enormous sample sizes would be required given the extreme variability of indications and cancer therapies and the assumed moderate effects. Moreover, prospective registry studies could hamper recruitment into properly designed clinical trials.

As patients will continue demanding methadone for cancer, it is now time to initiate randomized controlled clinical trials with methadone in cancer patients. Doxorubicin could be used in a 2x2 design or as a factor in the analysis, calling for patients eligible for doxorubicin therapy. Instead, cancer pain should be no selection criterion. The control group could be placebo; however, a reasonable alternative could be watchful waiting for, say, 6 months. This is because most “active” patients and some investigators might correctly guess the true nature of blinded treatment. Moreover, open designs would facilitate treatment of breakthrough pain, maybe with other opioids. Anyhow, the focus should be on opioid-naïve patients.

The dosage of methadone could be based on the paper by Onken *et al*. ^47^. A dose or concentration controlled design could be considered ^53^. To investigate opioid-experienced patients, a randomized withdrawal design ^54^ might be an option, initially switching all patients to methadone and then withdrawing (or not) it in a blinded manner after, say, 2 weeks. Under such circumstances, it would be unwise to administer any chemotherapy before the end of the withdrawal phase.

Under these prerequisites, methadone appears to be sufficiently safe for initiating a large trial. It should be stressed that it is unclear as to whether methadone would “win” against control. However, almost any outcome would relevantly expand medical knowledge and provide appropriate arguments for answering patients’ hopes, whether justified or not.

## Conclusions

- Cancer patients are asking for a methadone therapy, although such therapy is not yet supported by clinical evidence.
- There are nonclinical data supporting its usefulness against certain types of cancer or as enhancer of doxorubicin.
- Strong epidemiologic data support its usefulness for cancer, while alternative explanations rather appear unlikely.
- It is time to initiate randomized controlled clinical trials to test the efficacy of methadone as a therapy for cancer patients.

## Data availability

No data are associated with this article.

## Author endorsement

Wolfgang Bessler confirms that the author has an appropriate level of expertise to conduct this research, and confirms that the submission is of an acceptable scientific standard. Wolfgang Bessler declares they have no competing interests. Affiliation: Institute of Molecular Medicine and Cell Research, University of Freiburg, Freiburg im Breisgau, Germany
