# Supplementary material for: Time to initiate randomized controlled clinical trials with methadone in cancer patients
Source: F1000Res. 2022 May 12;8:1835. Originally published 2019 Oct 31. [Version 2] doi: 10.12688/f1000research.20454.2 (PMC9091806; doi:10.12688/f1000research.20454.2)
Supplement: Peer review history

# Peer review: Reviewer response for version 1

**DOI:** 10.5256/f1000research.22485.r136917

**Published:** 2022-05-10

**Authors:** Harald Köfeler

**Affiliations:** 1 Core Facility Mass Spectrometry, Medical University of Graz, Graz, Austria

The opinion article titled ‚Time to initiate randomized controlled clinical trials with methadone in cancer patients‘ by HJ Kremer is a timely topic with high relevance in the field of cancer therapy. The existing literature about methadone use in cancer therapy is comprehensively evaluated ranging from preclinical findings to epidemiological studies. While the data are not conclusive at this stage, there is reasonable hope that better study designs could provide conclusive data. In fact, many studies are either too small in size or do not have an appropriate study design to be useful. In this sense, the author provides valuable ideas for the design of future clinical trials judging the use of methadone in cancer therapy. At least the reviewed data at hand as of today are encouraging to further pursue this anti-cancer strategy because if future findings are negative there is not much lost, but if they are positive it could be a significant step forward. In summary, this manuscript could be an incentive for the scientific community to pick up an important topic in cancer therapy, which has so far been severely neglected.

**Minor comments:**

- Page 2, paragraph 2, line 14: ‘…all the…’ instead of ‘…the all’.
- Page 2, paragraph 3, line 6: ‘…unless otherwise…’

Is the topic of the opinion article discussed accurately in the context of the current literature?

Yes

Are arguments sufficiently supported by evidence from the published literature?

Yes

Are all factual statements correct and adequately supported by citations?

Yes

Are the conclusions drawn balanced and justified on the basis of the presented arguments?

Yes

Reviewer Expertise:

Biochemistry

I confirm that I have read this submission and believe that I have an appropriate level of expertise to confirm that it is of an acceptable scientific standard.

# Peer review: Reviewer response for version 1

**DOI:** 10.5256/f1000research.22485.r66075

**Published:** 2020-08-10

**Authors:** Mary Lynn McPherson

**Affiliations:** 1 University of Maryland, Baltimore, MD, USA

I think this is an excellent paper that offers a substantiated proposal that it is indeed time to initiate a randomized controlled clinical trial in cancer patients to determine if methadone therapy improves outcomes or mortality data. The public has become increasingly well-informed (thank you Dr. Google) and the idea that methadone may improve longevity in cancer is too titillating to ignore. However, the data is often controversial and by no means conclusive. Kremer does an excellent job reviewing what we do know to date on this topic and affirms that we are not in a position to support the uncontrolled distribution of methadone for this purpose. He does, however, suggest the time has come to “initiate randomized controlled clinical trials with methadone in cancer patients.”

**Additional comments:**

 In more detail, Kremer provides the background on how the general public has become aware of the potential for methadone possibly improving outcomes in cancer patients. This has resulted in patients demanding methadone therapy when diagnosed with cancer, regardless of the presence of pain. A chronological history of preclinical findings (predating clinical observations) follows, dating back to the 1980s. Interestingly, high concentrations of methadone (but not other opioids) boosted the effect of vinblastine in multidrug resistant cells. Research groups from Germany, Spain, and Canada found that methadone induced apoptosis in cancer cells and was able to overcome chemotherapy resistance.

 Survival benefit was explored in several studies. The largest included almost 100,000 patients in the Veterans Affairs system. A hazard ratio of 0.56 was seen for survival benefit with methadone-treated patients vs. long-acting morphine in a cohort with non-metastatic cancer, non-HIV, and non-addicts. Several other studies also showed positive outcomes with methadone, although a retrospective cohort study from a Tennessee Medicaid database showed increased mortality with methadone. Two targeted investigations also showed beneficial effects with methadone, although they were small in size.

 Kremer concludes by asking more questions than he answers? Is methadone safer or provides benefits in cancer over other opioids? Going on the assumption that there may be something to the protective or beneficial effects of methadone in certain cancers, and possibly as an enhancer of doxorubicin. I agree with Dr. Kremer that it is “time to initiate randomized controlled clinical trials to test the efficacy of methadone as a therapy for cancer patients.”

Is the topic of the opinion article discussed accurately in the context of the current literature?

Yes

Are arguments sufficiently supported by evidence from the published literature?

Yes

Are all factual statements correct and adequately supported by citations?

Yes

Are the conclusions drawn balanced and justified on the basis of the presented arguments?

Yes

Reviewer Expertise:

Palliative care, medication management, opioid conversion calculations, methadone dosing

I confirm that I have read this submission and believe that I have an appropriate level of expertise to confirm that it is of an acceptable scientific standard.
